# Poster Session II - A246 DIETARY TRACKING REVEALS PERSONALIZED DIET RESPONSES LINKED TO GUT INFLAMMATION AND MICROBIOME VARIATION IN CROHN’S DISEASE FIRST-DEGREE RELATIVES

**DOI:** 10.1093/jcag/gwaf042.246

**Published:** 2026-02-13

**Authors:** I Shah, A Waslyk, R Murphy, M Xue, J Shao, B Bharali, Q Li, C Dang, H Leibovitzh, S Lee, K Croitoru, W Turpin

**Affiliations:** Lunenfeld-Tanenbaum Research Institute, Toronto, ON, Canada; Lunenfeld-Tanenbaum Research Institute, Toronto, ON, Canada; North Simcoe Family Health Team, Midland, ON, Canada; Lunenfeld-Tanenbaum Research Institute, Toronto, ON, Canada; Lunenfeld-Tanenbaum Research Institute, Toronto, ON, Canada; Lunenfeld-Tanenbaum Research Institute, Toronto, ON, Canada; Lunenfeld-Tanenbaum Research Institute, Toronto, ON, Canada; University of Toronto, Toronto, ON, Canada; Sinai Health, Toronto, ON, Canada; Lunenfeld-Tanenbaum Research Institute, Toronto, ON, Canada; Lunenfeld-Tanenbaum Research Institute, Toronto, ON, Canada; Lunenfeld-Tanenbaum Research Institute, Toronto, ON, Canada

## Abstract

**Background:**

Although diet is a key modifiable risk factor for Crohn’s disease (CD), the extent of individual variability in dietary effects remains poorly understood.

**Aims:**

This study addressed this gap by integrating picture-based dietary monitoring with fecal calprotectin (FCP) measurements to investigate personalized dietary influences on CD risk biomarkers and the gut microbiome.

**Methods:**

We conducted a 7-week longitudinal study in healthy first-degree relatives (FDRs) of CD patients to assess personalized dietary effects on FCP and gut microbiome dynamics. Participants recorded 3–5 daily meals using RxFood, an app that applies convolutional neural network analysis to food pictures to generate dietary assessments. FCP was quantified by ELISA, and diet–FCP associations were captured by mixed-effects Bayesian network (MEBN) model. K-means clustering analysis revealed distinct, individualized patterns across nutrient predictors. Gut microbiome profiling was performed by shotgun metagenomic sequencing and processed to genus level using *HUMAnN3* pipeline. Microbiome differences across diet-FCP response clusters were assessed using Kruskal-Wallis, Wilcoxon, PCoA, and PERMANOVA.

**Results:**

The study analyzed data from 23 participants, including 13,753 food items from 6,616 meals and 682 stool samples. Using an MEBN model, 55% of the dietary components showed consistent associations with FCP with cholesterol positively and fiber negatively associated, while the remaining dietary components displayed individual-specific effects. Clustering analysis of personalized nutrient-FCP estimates revealed four distinct clusters, each characterized by unique nutrient-FCP associations. These clusters also showed significant differences in gut microbiome composition, including genus-level variations (*Faecalibacterium*, *Bacteroides*, *Roseburia*, *Ruminococcus*, *Collinsella*) and distinct community structures (PERMANOVA, p = 0.001).

**Conclusions:**

Dietary monitoring using image recognition provides a practical and objective approach to assess nutrition in individuals at risk for CD. Our findings demonstrate that diet has personalized effects on FCP levels, and that these dietary response patterns are accompanied by distinct differences in gut microbiome composition. Together, these results highlight the potential for developing tailored dietary interventions to modulate intestinal inflammation and reduce the risk of CD.

**Funding Agencies:**

Weston family foundation

